# Dose-response meta-analysis: application and practice using the R software

**DOI:** 10.4178/epih.e2019006

**Published:** 2019-03-28

**Authors:** Sung Ryul Shim, Jonghoo Lee

**Affiliations:** 1 Department of Preventive Medicine, Korea University College of Medicine, Seoul, Korea; 2 Urological Biomedicine Research Institute, Soonchunhyang University Hospital, Seoul, Korea; 3 Department of Internal Medicine, Jeju National University Hospital, Jeju National University School of Medicine, Jeju, Korea

**Keywords:** Meta-analysis, Dose response, Quadratic model, Restricted cubic spline, Nonlinearity, Dosresmeta

## Abstract

The objective of this study was to describe the general approaches of dose-response meta-analysis (DRMA) available for the quantitative synthesis of data using the R software. We conducted a DRMA using two types of data, the difference of means in continuous data and the odds ratio in binary data. The package commands of the R software were “doseresmeta” for the overall effect sizes that were separated into a linear model, quadratic model, and restricted cubic split model for better understanding. The effect sizes according to the dose and a test for linearity were demonstrated and interpreted by analyzing one-stage and two-stage DRMA. The authors examined several flexible models of exposure to pool study-specific trends and made a graphical presentation of the dose-response trend. This study focused on practical methods of DRMA rather than theoretical concepts for researchers who did not major in statistics. The authors hope that this study will help many researchers use the R software to perform DRMAs more easily, and that related research will be pursued.

## INTRODUCTION

The basic purpose of dose-response meta-analysis (DRMA) is to reveal the relationship between disease risk and exposure dose [1-3].

If there are multiple effect sizes according to the doses of medicine when statistics are extracted in studies for meta-analysis, the researcher may be puzzled about whether to analyze only one effect size or the effect sizes of all doses in one study. If he or she chooses the former, this may result in selection bias even before the research is begun; if the latter is chosen, there is no adequate meta-analysis method.

The most appropriate statistical method in this case is DRMA. It is particularly useful because it can show the trend of risks according to the exposure dose when the risks of exposure doses are presented as three or more categorical data [3].

Recently, various tools for DRMA were developed. In particular, the “dosresmeta” package of R software and the “drmeta” of STATA software using the two-stage method developed by Nicola Orsini can analyze even continuous data that could not be dealt with in the past [4].

In this study, previous meta-analysis studies performed by the authors [1-3] are reviewed using the R software. Furthermore, this study requires prior knowledge about DRMA because it focuses on using the basic commands of R and plotting methods required for performing DRMA.

## UNDERSTANDING DOSE-RESPONSE METAANALYSIS

### Dose-response meta-analysis model

Greenland & Longnecker [5] proposed a method of estimating linear trends using generalized least-square regression to correlate between the referent group and risk at each exposure dose level. Furthermore, Berlin et al. [6] proposed a two-stage meta-analysis method that applied a random effect regression model to multiple dose-response studies.

A two-stage meta-analysis consists of obtaining the regression coefficient of each individual study in the first stage, and calculating the total coefficient by converging the weighted averages of the regression coefficients of individual studies in the second stage. However, this method is not applicable to non-linear relationships, although it can be applied when a linear trend is assumed [3].

Therefore, an analysis method that assumes non-linearity is required, and an analysis method that assumes a non-linear trend when examining the effect size for each dose level was developed [7]. Liu et al. [8] suggested a method of applying a quadratic random effect model that converges well even in a non-linear relationship, and Orsini & Greenland [9] discretized a flexible nonlinear model with restricted cubic splines (RCS). Consequently, the current standardized DRMA method statistically tests for non-linearity and shows it graphically [1-3].

Among the models that are dependent on time and dose, nonlinear models such as the quadratic model and RCS model generally tend to reflect phenomena better than linear models [2]. Therefore, when performing a first analysis, efforts should be made to find the most appropriate model for the research after sufficiently reviewing various models.

### Determining the exposure dose

There are few dose-response studies that accurately present specific doses. Most of them present doses as medians or ranges. Doses presented as medians can be used as they are, but the ranges must be converted to specific doses.

The basic method is as follows:

First, the dose of the reference group is zero (e.g., when the odds ratio [OR] is 1). Second, if the beginning of the smallest dose group is open, then the median of the corresponding group is set as the dose assuming that the beginning is zero (e.g., 5 if <10). Third, if the beginning and end of a dose group are closed by specific values, then the median is set as the dose (e.g., 15 if 10-20). Fourth, if the end of the largest dose group is open, the value of the median of the previous dose group minus the beginning value of the previous dose group (e.g., 15-10=5) is added to the beginning value of the last group, and the resulting value is set as the dose (e.g., 25 if >20) [1-3].

## DOSE-RESPONSE META-ANALYSIS USING “DOSRESMETA” PACKAGE OF R

**Figure 1.**
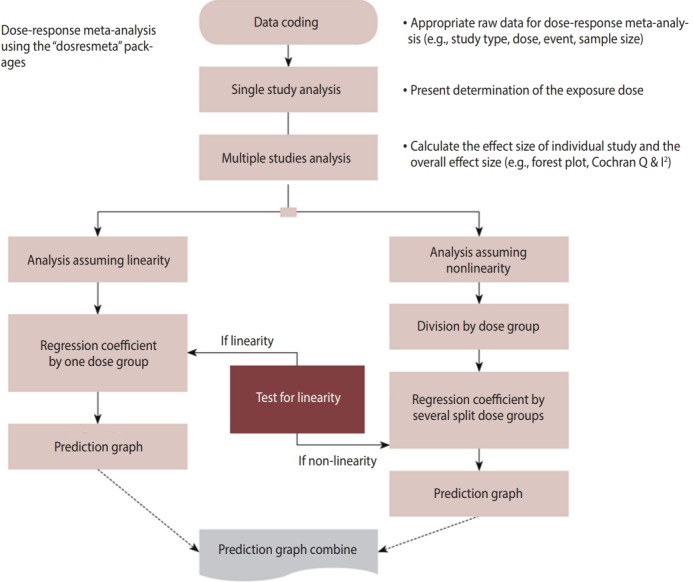
Flowchart of dose-response meta-analysis using the R “dosresmeta” package.

Figure 1 shows the flow of a DRMA. When coding the first data item, the variable names must be changed appropriately for the corresponding function. An analysis that assumes linearity and an analysis that assumes non-linearity are performed simultaneously, and then the result is reported.

The “dosresmeta” package is needed to perform DRMA in R. Furthermore, the “mvtnorm,” “ellipse,” and “mvmeta” packages related to multivariate meta-analysis and the “rms” package for a RCS must also be installed in advance, as follows:

· install.packages(“dosresmeta”)

· install.packages(“mvtnorm”)

· install.packages(“ellipse”)

· install.packages(“mvmeta”)

· install.packages(“rms”)

In addition, you should install the “meta,” “metafor,” and “rmeta” packages for general intervention meta-analysis in R, as follows:

· install.packages(“meta”)

· install.packages(“metafor”)

· install.packaqes(“rmeta”)

For detailed explanations about DRMA, you can refer to the detailed codes, documents, and references for each package [4].

We mark R commands with a dot (‘· ’) in front of them, to distinguish them from the main text. When long commands are extended to the next line, there is no dot at the beginning of the next line. Thus, when you enter the command in the R software, you must type them without the dot (‘· ’) in front of them.

### Binary data example

#### Data coding and loading

As example binary data for DRMA, the risk of cardiovascular disease (CVD) according to alcohol intake was extracted from the example data of R (Supplementary Material 1).

One thing to note when coding data is to distinguish studies by id numbers. In addition, for type, you must enter cc, ir, or ci for case control, incidence rate, or cumulative incidence, respectively.

Load the example file to the memory of R from the working folder by using the following command. One thing to note is that R prefers the comma-separated value (csv) file format. Thus, you should save the data in Supplementary Material 1 as “drma_bin.csv” in the specified working folder.

·data_bin <- read.csv(“drma_bin.csv”, header=TRUE)

“read.csv” is a function for loading a csv file. The above command means to load the file “drma_bin.csv” and use the first variable name of the file (header=TRUE). This loaded file is saved as “data_bin” in the R memory.

### Scatter plot graph for raw data

Before starting the model analysis, we will draw a scatter plot to see the overall outline of the example file.

This is easier to understand intuitively if we use the inverse number of the standard error (SE) as a weight in the example binary data. Hence, the reverse SE variable is additionally created in the data_bin data.

·data_bin$inver_se <- 1/data_bin$se

For the graph, the “ggplot2” package is generally used.

· library(ggplot2)

· ggplot(data_bin, aes(dose, logrr, size=inver_se)) + geom_point(shape=1, colour=“black”) + scale_size_area(max_size=20)

After loading the “ggplot2” package in the memory, sequentially enter the above arguments. There are three groups in total. The first ggplot part is the background of the graph, and geom_point and scale_size_area are options for appropriately expressing individual graphs.

Enter the data name in the first parentheses for ggplot, and enter the horizontal axis variable (dose), vertical axis variable (logrr), and point variable (inver_se) in the “aes” function. The following parts set the size, color, and so on of the corresponding points. You can easily understand the individual elements by performing simulations several times.

**Figure 2.**
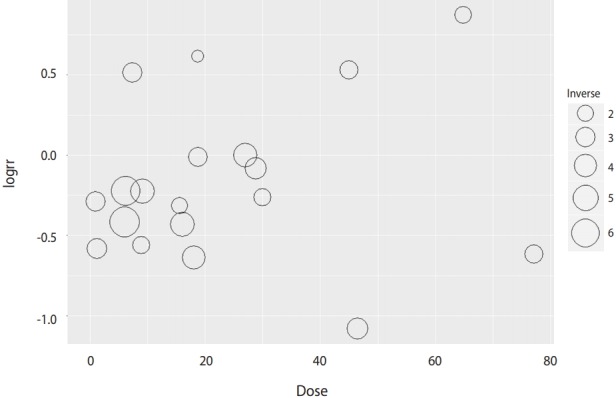
Scatter plot of binary sample data.

Figure 2 shows a scatter plot for the dose and effect size (logrr). You can see the effect size for an individual dose. The size of the circle is the inverse number of the SE. Thus, a larger circle indicates a more accurate and better study.

All of the effect sizes have an increasing trend except for the two effect sizes at the bottom right. Thus, we will test them using various analysis models.

#### Linear model

To perform a DRMA, load the “dosresmeta” package:

· library(dosresmeta)

An analysis that assumes linearity is performed by treating the dose variables as a bundle.

Each of the collected studies has one reference group. The command for calculating the total regression coefficient after converging the weighted averages of the regression coefficients of individual studies using the two-stage method is as follows:

· lin_bin <- dosresmeta(formula=logrr ~ dose, id=id, type=type, se=se, cases=cases, n=n, data=data_bin)

· summary(lin_bin)

The linear model lin_bin is created by entering various arguments in the “dosresmeta” function. “formula” functions as a type of regression analysis in the DRMA. Hence, enter the dependent variable logrr after the formula, and the independent variable dose after “~”. Next, enter id, type, se, cases, and n. Then, set the data_bin with these variables.

To examine the results of the summary command in the console window, this analysis is based on a two-stage method developed by Nicola Orsini, the restricted maximum likelihood (REML) model, and uses the method of Greenland & Longnecker [5] for covariance.

The estimated regression coefficient is -0.0044, which is statistically insignificant (p=0.4585).

Furthermore, the p-value (0.0147) and I^2^ value (64.7%) of the Cochrane Q statistics are shown, which indicate heterogeneity. Thus, it can be seen that this linear model has heterogeneity.

The estimated regression coefficient is exponential-transformed for interpretation because it was log-transformed.

· predict(lin_bin, delta=1, exp=TRUE)

After the exponential transformation, the risk is 0.996 (95% confidence interval [CI], 0.984 to 1.007). To interpret this, the risk of CVD increases by 0.996 times when one unit dose of alcohol is ingested (or decreases by 0.4%). However, this was statistically insignificant because the 95% CI includes 1.000.

### Linear model graph

**Figure 3.**
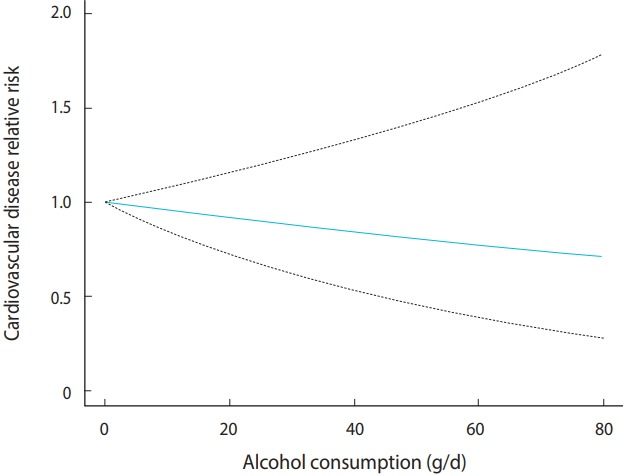
Linear model of binary sample data.

The linear model is plotted in Figure 3.

· dosex_bin <- data.frame(dose=seq(0, 80, 1))

· with(predict(lin_bin, dosex_bin, order=TRUE, exp=TRUE), {plot(dose, pred, type=“l”, col=“blue”, ylim=c(0, 2), ylab=“cardiovascular disease relative risk”, xlab=“alcohol consumption, grams/day”)

lines(dose, ci.lb, lty=2)

lines(dose, ci.ub, lty=2)})

The x-axis of the graph, dosex_bin, is set by making the dose from 0 to 80 as one unit with the “data.frame” function. This is also used as the x-axis in the following quadratic model and cubic spline model.

The graph-drawing commands must be executed by using the entire “with” part at once.

In the “with” part, you can directly specify the reference data for creating a graph. In the “predict” function, sequentially enter the linear analysis model (lin_bin), the x-axis of the graph (dosex_bin), and exponential transformation (exp=TRUE). In the final analysis, the “with” part means to draw a graph with the predicted values of the regression model obtained by entering the dose from 0 to 80 as one unit in the linear analysis model.

Next, the “plot” function draws the actual graph corresponding to the dose on the x-axis and pred on the y-axis. The other commands are options to improve the look of the graph. You can learn their meanings by arbitrarily adding or removing them.

ylim=c(0, 2) indicates the y-axis only between 0 and 2. You should adjust this value appropriately to express the graph properly. The last two lines are commands to add lines for a 95% CI.

As with the interpretation of the regression coefficient of the linear model, the risk decreases with the dose. The blue solid line in the middle indicates the risk of CVD, and the upper and lower dotted lines indicate the 95% CI (Figure 3).

#### Quadratic model

Now, we will create a quadratic model that does not assume perfect linearity. One simple method is to square-transform the dose.

The method of calculating the regression coefficient is the same as for the abovementioned linear model. When the model is established, the dose and dose square transformation are added to the independent variables.

· quad_bin <- dosresmeta(formula=logrr ~ dose + I(dose^2), id=id, type=type, se=se, cases=cases, n=n, data=data_bin)

· summary(quad_bin)

The “dosresmeta” function is used. Enter the dependent variable, logrr, after formula, and place the independent variables of dose and square of the dose after “~”. Then, enter id, type, se, cases, and n, and set the data_bin with these variables. This model is set as quad_bin.

The total model is statistically significant with a p-value of 0.0414.

The regression coefficient of the estimated dose is -0.0302, which is statistically significant (p=0.022). Furthermore, the p-value (0.0001) and I^2^ value (71.5%) of the Cochrane Q statistics are shown, which indicate heterogeneity. Thus, it can be seen that this model has heterogeneity.

The estimated regression coefficient is exponential-transformed for interpretation because it was log-transformed.

· exp(-0.0302)

The risk after exponential transformation is 0.970. To interpret this, when 1 unit dose of alcohol is ingested, the risk of CVD increases by 0.970 times (or decreases by 3%). This is statistically significant.

### Quadratic model graph

**Figure 4.**
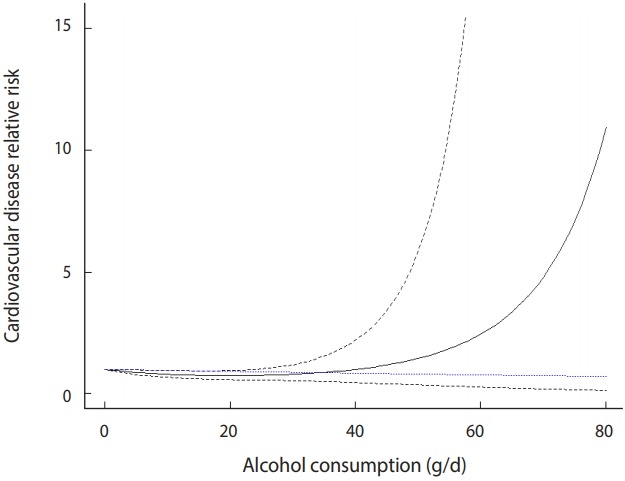
Quadratic model of binary sample data.

The quadratic model is plotted in Figure 4.

· with(predict(quad_bin, dosex_bin, exp=TRUE), {plot(dose, pred, type=“l”, ylim=c(0, 15), ylab=“cardiovascular disease relative risk”, xlab=“alcohol consumption, grams/day”)

lines(dose, ci.lb, lty=2)

lines(dose, ci.ub, lty=2)})

· points(dosex_bin$dose, predict(lin_bin, dosex_bin, exp=TRUE)$pred, type=“l”, lty=3, col=“blue”)

The explanation of the graph command is the same as for the linear model.

However, ylim=c(0, 15) means that the y-axis is displayed only between 0 and 15. These values must be adjusted appropriately to represent the graph properly.

The last points command displays the linear model in the same graph.

The black solid line in the middle indicates the risk, and the upper and lower black dotted lines indicate a 95% CI. The blue dotted line at the bottom indicates the risk of the linear model.

The risk in the quadratic model is slightly different from the risk in the linear model. It is similar until a dose of approximately 40, but the risk tends to increase after that and increases sharply from 70. Therefore, according to the quadratic model, when the alcohol intake dose is high (higher than 40), the risk of CVD increases sharply (Figure 4).

#### Restricted cubic spline model

We will create a cubic spline model among the non-linear models. The method of calculating the regression coefficient is the same as in the abovementioned linear model, but the dose level must be subdivided beforehand.

■ Division of dose level

The dose levels must be subdivided to examine the risk of a dose-response at each level. What should be noted here is that the doses of individual studies are not the same, and the level setting of the dose group can be a problem because the relationship of the risk to the dose-response is non-linear. Therefore, the best approach to the dose group setting in a non-linear relationship that has been developed until now is to subdivide the levels using RCS [1-3].

· library(“rms”)

· knots_bin <- quantile(data_bin$dose, c(.05, .35, .65, .95))

In R, RCS can be used through the “rms” package. After specifying data in the quantile function, the 5%, 35%, 65%, and 95% intervals are divided as vectors. The number and range of the dose levels can be adjusted appropriately. When you set four doses, a total of three dose levels are created for knots_bin (knots-1).

■ Calculation of dose level risk (regression coefficient)

· spl_bin <- dosresmeta(formula=logrr ~ rcs(dose, knots_bin), type=type, id=id, se=se, cases=cases, n=n, data=data_bin)

· summary(spl_bin)

The “dosresmeta” function is used. Enter the dependent variable, logrr, in the formula. For independent variables, enter the dose and the previously divided three dose levels after “~” in the RCS function. Then, enter ID, type, se, cases, and n, and set the data_bin with these variables. This model is set as spl_bin.

The goodness of fit of this model is statistically significant (p=0.038).

The estimated regression coefficient for each dose level can be verified, but interpreting the risk for each dose level is meaningless because the dose levels were arbitrarily divided to test the linearity.

■ Testing the linearity of dose level risk (regression coefficient)

The most important part in an analysis that assumes non-linearity is to test whether the slopes of the regression lines for each dose level have statistically significant differences. If they do not show significant differences, it can be determined that there is linearity.

In an analysis that assumes non-linearity, the differences in the slopes of the three dose levels can be tested as follows:

· waldtest(b=coef(spl_bin), Sigma=vcov(spl_bin), Terms=2:3)

The command for testing linearity is “waldtest”. The first of the three dose levels is excluded because it is the total raw data itself, and the joint slope of the second and third dose levels is tested.

The null hypothesis (H0): doses2=doses3=0.

If the p-value is larger than 0.05, the null hypothesis is accepted, and the joint slope is zero, meaning that there is no slope. Because the slopes of the two dose levels have no difference, it is determined that the model has linearity.

If the p-value is smaller than 0.05, the null hypothesis is rejected, and the joint slope is not zero. Thus, because there is a slope or the slopes of the two dose levels have a difference, it is determined that the model has nonlinearity.

As a result of waldtest, the p-value is 0.018. Therefore, this model has non-linearity.

- Cubic spline model graph

**Figure 5.**
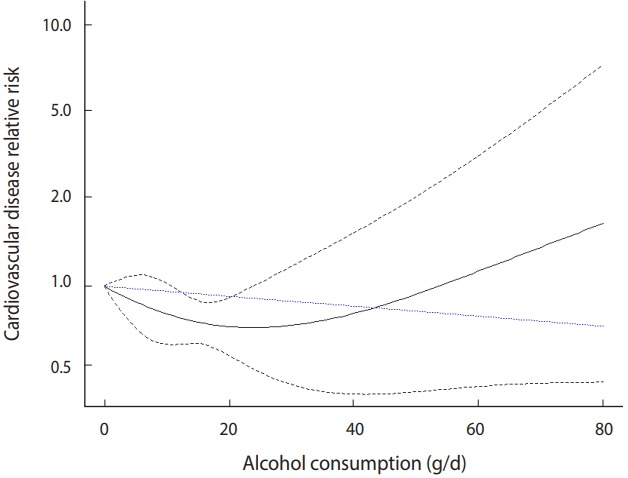
Restricted cubic spline model of binary sample data (reference = 0).

The cubic spline model is plotted in Figure 5.

· xref_bin <- 0

· with(predict(spl_bin, dosex_bin, xref_bin, exp=TRUE),{plot(get(“rcs(dose, knots_bin)dose”), pred, type=“l”, ylim=c(0.4, 10), ylab=“cardiovascular disease relative risk”, xlab=“alcohol consumption, grams/day”, log=“y”, bty=“l”, las=1) matlines (get(“rcs(dose, knots_bin)dose”), cbind(ci.ub, ci.lb), col=1, lty=“dashed”)})

· points(dosex_bin$dose, predict(lin_bin, dosex_bin, xref_bin, exp=TRUE)$pred, type=“l”, lty=3, col=“blue”)

The explanation for the graph command is the same as in the linear model. xref_bin specifies a certain value for the dose reference variable. If you change the value of the reference variable appropriately according to the shape of the created graph, your discriminating ability will improve. Hence, you can set the value to zero at first and change it according to the shape of the graph.

ylim=c(0.4, 10) means that the y-axis is displayed only between 0.4 and 10. The values must be adjusted appropriately to represent the graph properly. The last “points” command displays the linear model in the same graph.

The black solid line in the middle indicates the risk, and the upper and lower black dotted lines indicate a 95% CI. The blue dotted line at the bottom indicates the risk of the linear model.

The risk in the cubic spline model is slightly different from the risk in the linear model. It is similar until a dose of approximately 25, but the risk tends to increase after that and increases sharply from 40. Therefore, according to the cubic spline model, when the alcohol intake dose is high (higher than 30), the risk of CVD increases sharply (Figure 5).

- Adjusting the cubic spline model graph

In Figure 5, the width of the 95% CI begins to decrease at a dose of approximately 17 and is recognized as an inflexion point. This value can be checked as follows:

· pre_bin <- predict(spl_bin, dosex_bin, exp=TRUE)

· pre_bin$ci.ub - pre_bin$ci.lb

When you calculate the width of the CI after obtaining all of the predicted values of the RCS model, you can see that the width is the smallest at the 17th dose, which is 0.2679, in the console window. Thus, the graph can be drawn again by setting this as the inflection point, as follows:

· xref_bin <- 17

**Figure 6.**
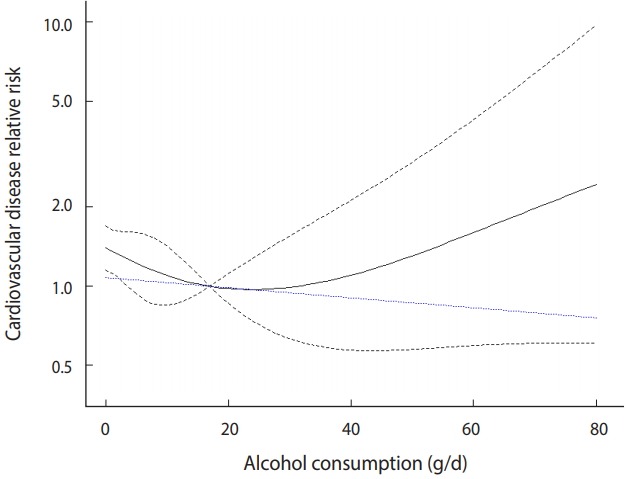
Restricted cubic spline model of binary sample data (reference = 17).

When the command for creating the cubic spline model graph is performed after changing the dose reference variable to 17, the graph in Figure 6 is obtained.

After changing the value of the reference variable, Figure 6 shows a J shape, which rises at the left and right around the dose of 17. Therefore, more intuitive interpretation is possible.

### Continuous data example

A continuous data example for the DRMA was extracted from the example data of R for the positive and negative syndrome scale, which measures the degree of schizophrenia according to the medicine dose (Supplementary Material 1). The commands for meta-analysis are almost identical to those in the binary data example. Refer to the reference [2] and appedix commands for detailed descriptions for continuous data.

## CONCLUSION

This study minimized statistical theory and focused on the actual performance of meta-analysis so that general researchers who have not majored in statistics can easily understand it. In other words, this study aimed to allow general researchers to adequately use already developed statistical methods in their field of study and to interpret the results.

The R packages for DRMA can handle very powerful functions. Researchers can use these analysis methods once they familiarize themselves with the software. Furthermore, although it is not explained in this study, the “drmeta” command in STATA can be used. This is the same analysis tool.

Furthermore, we hope that this study will help researchers perform meta-analyses more easily and pursue related research.

## SUPPLEMENTARY MATERIALS

Korean version is available at http://www.e-epih.org/).
