# Supplementary material for: Dose-response meta-analysis: application and practice using the R software
Source: Epidemiol Health. 2019 Mar 28;41:e2019006. doi: 10.4178/epih.e2019006 (PMC6635664; doi:10.4178/epih.e2019006)
Supplement: Supplementary file 1 [file epih-41-e2019006-supplementary.pdf]

Supplementary Material 1. R 용량-반응 메타분석 코드

```
#예제자료는 drma_bin.csv(Table S1), drma_con.csv(Table S2) 임.

#“dosresmeta” 패키지 불러오기
library("dosresmeta")
```

R의 “doseresmeta” 패키지를 이용한 용량-반응 메타분석

```
#데이터 코딩 및 불러오기
dta_shim <- read.csv("dta_shim.csv", header=TRUE)
```

▶ BINARY DATA

```
#데이터 코딩 및 불러오기
data_bin <- read.csv("drma_bin.csv", header=TRUE)

##################################### 원자료 산점도 그래프
library(ggplot2)
###### 최초 연구들의 효과크기를 역표준오차의 크기로 개괄적으로 보여줌.
data_bin$inver_se <- 1/data_bin$se #se의 역수를 만듬(동그라미가 클수록 좋은연구)

#최초 연구의 분포를 보여줌 (개별연구별 참조변수는 제외된다)
ggplot(data_bin, aes(dose, logrr, size=inver_se)) +     geom_point(shape=1, colour="black")
        +     scale_size_area(max_size=20)
####################################

###Linear model###
lin_bin <- dosresmeta(formula = logrr ~ dose, id = id, type = type, se = se, cases = cases,
        n = n, data = data_bin)
summary(lin_bin)
predict(lin_bin, delta = 1, exp = TRUE)

#Graphical results
dosex_bin <- data.frame(dose = seq(0, 80, 1)) #그래프 x축을 용량을보고 설정해놓는다.
with(predict(lin_bin, dosex_bin, order = TRUE, exp = TRUE), {
   plot(dose, pred, type = "l", col = "blue", ylim = c(0, 2),
         ylab = "cardiovascular disease relative risk", xlab = "alcohol consumption,
        grams/day")
   lines(dose,   ci.lb, lty = 2)
   lines(dose, ci.ub, lty = 2)
})


###Quadratic model for Non-linear model ###
quad_bin <- dosresmeta(formula = logrr ~ dose + I(dose^2), id = id, type = type, se = se,
        cases = cases, n = n, data = data_bin)
```

```
summary(quad_bin)
predict(quad_bin, exp = TRUE)

#Graphical results
with(predict(quad_bin, dosex_bin, exp = TRUE), {
  plot(dose, pred, type = "l", ylim = c(0, 15),
       ylab = "cardiovascular disease relative risk", xlab = "alcohol consumption,
       grams/day")
  lines(dose,  ci.lb, lty = 2)
  lines(dose, ci.ub, lty = 2)
})
points(dosex_bin$dose, predict(lin_bin, dosex_bin, exp = TRUE)$pred, type = "l", lty = 3, col
       = "blue")


###Restricted cubic spline for Non-linear model###
library("rms") # restricted cubic spline을 사용할수있게해준다.
knots_bin <- quantile(data_bin$dose, c(.05, .35, .65, .95))
# *****만약 안돌아가면 vector갯수를(spline 개수를) 조절해볼것*****.
spl_bin <- dosresmeta(formula = logrr ~ rcs(dose, knots_bin), type = type, id = id, se = se,
       cases = cases, n = n, data = data_bin)
summary(spl_bin)

#선형성검정
waldtest(b = coef(spl_bin), Sigma = vcov(spl_bin), Terms = 2:3)

#Graphical results
xref_bin <- 0 #곡선의 모양에 따라서 ref를 변경해줄수있다. 처음에는 0으로 해보고 그래프
       의 모양에 따라 ref바꾸어보자
with(predict(spl_bin, dosex_bin, xref_bin, exp = TRUE),{
  plot(get("rcs(dose, knots_bin)dose"), pred, type = "l", ylim = c(0.4, 10),
       ylab = "cardiovascular disease relative risk", xlab = "alcohol consumption,
       grams/day",
       log = "y", bty = "l", las = 1)
  matlines(get("rcs(dose, knots_bin)dose"), cbind(ci.ub, ci.lb), col = 1, lty = "dashed")
})
points(dosex_bin$dose, predict(lin_bin, dosex_bin, xref_bin, exp = TRUE)$pred, type = "l", lty
       = 3, col = "blue")


######모양을 예쁘게 하기위한 첨가#######
pre_bin <- predict(spl_bin, dosex_bin, exp = TRUE)
pre_bin$ci.ub - pre_bin$ci.lb #17번째 값 0.2679로서 가장 폭이 좁다. 따라서 위의 그래프
       에서 xref <-17로 설정해서 재실행

############################
##waldtest##
#H0 : doses2=doses3=o
#P>0.05, doses2와 doses3의 조인트기울기는 0으로 기울기가 없으며 그리고 두 용량범주
       의 기울기는 차이가없다.
#따라서 선형성으로 판단한다.
#P<0.05, doses2와 doses3의 조인트기울기는 0이 아니어서 기울기가 있거나 또는 두 용
       량범주의 기울기는 차이가있다.
#따라서 비선형성으로 판단한다.
############################
```

▶ CONTINUOUS DATA

```
#데이터 코딩 및 불러오기
data_con <- read.csv("drma_con.csv", header=TRUE)

###Linear model###
lin_con <- dosresmeta(formula = y ~ dose, id = id, sd = sd, n = n, covariance = "smd", data
        = data_con)
summary(lin_con)

#Graphical results
dosex_con <- data.frame(dose = seq(0, 30, 1)) #그래프 x축을 용량을보고 설정해놓는다.
with(predict(lin_con, dosex_con, order = TRUE), {
  plot(dose, pred, type = "l", col = "blue", ylim = c(0, .6),
        ylab = "schizophrenia score", xlab = "medicine dose level, mg/day")
  lines(dose,  ci.lb, lty = 2)
  lines(dose, ci.ub, lty = 2)
})


###Quadratic model for Non-linear model ###
quad_con <- dosresmeta(formula = y ~ dose + I(dose^2), id = id, sd = sd, n = n, covariance
        = "smd", data = data_con)
summary(quad_con)

#Graphical results
with(predict(quad_con, dosex_con, order = TRUE), {
  plot(dose, pred, type = "l", ylim = c(0, .6),
        ylab = "schizophrenia score", xlab = "medicine dose level, mg/day")
  lines(dose,  ci.lb, lty = 2)
  lines(dose, ci.ub, lty = 2)
  rug(dose, quiet = TRUE)
})
points(dosex_con$dose, predict(lin_con, dosex_con)$pred, type = "l", lty = 3, col = "blue")


###Restricted cubic spline model for Non-linear analysis###
library("rms") # restricted cubic spline을 사용할수있게해준다.
knots_con <- quantile(data_con$dose, c(.05, .5, .95))
# *****만약 안돌아가면 vector갯수를(spline 개수를) 조절해볼것*****.
spl_con <- dosresmeta(formula = y ~ rcs(dose, knots_con), id = id, sd = sd, n = n, covariance
        = "smd", data = data_con)
summary(spl_con)

#선형성검정
waldtest(b = coef(spl_con), Sigma = vcov(spl_con), Terms = 1:2)

#Graphical results
xref_con <- 0 #곡선의 모양에 따라서 ref를 변경해줄수있다.
with(predict(spl_con, dosex_con, xref_con),{
  plot(get("rcs(dose, knots_con)dose"), pred, type = "l", ylim = c(0, .6),
        ylab = "schizophrenia score", xlab = "medicine dose level, mg/day", bty = "l", las =
```

```
        1)
  matlines(get("rcs(dose, knots_con)dose"), cbind(ci.ub, ci.lb), col = 1, lty = "dashed")
})
points(dosex_con$dose, predict(lin_con, dosex_con, xref_con)$pred, type = "l", lty = 3, col =
        "blue")
```

Table S1. The sample data for binary outcomes

author	id	type	dose	cases	n	logrr	se
Bianchi	1	cc	0.00	126	414	0.000	NA
Bianchi	1	cc	9.06	61	261	-0.223	0.223
Bianchi	1	cc	27.00	69	228	0.000	0.234
Bianchi	1	cc	45.00	22	44	0.531	0.377
Bianchi	1	cc	64.80	19	34	0.875	0.444
Bobak	2	cc	0.00	77	258	0.000	NA
Bobak	2	cc	16.05	88	413	-0.431	0.221
Bobak	2	cc	46.42	24	202	-1.079	0.298
Bobak	2	cc	77.16	13	64	-0.616	0.387
Malarcher-wine	3	cc	0.00	83	208	0.000	NA
Malarcher-wine	3	cc	1.18	46	175	-0.580	0.317
Malarcher-wine	3	cc	8.92	17	58	-0.562	0.419
Malarcher-wine	3	cc	18.72	4	11	0.615	0.909
Malarcher-beer	4	cc	0.00	83	208	0.000	NA
Malarcher-beer	4	cc	0.95	29	117	-0.288	0.333
Malarcher-beer	4	cc	7.43	32	81	0.513	0.338
Malarcher-beer	4	cc	15.60	18	39	-0.315	0.471
Vliegenthart-wine	5	ci	0.00	159	480	0.000	NA
Vliegenthart-wine	5	ci	6.00	229	975	-0.416	0.144
Vliegenthart-wine	5	ci	18.00	38	207	-0.635	0.238
Vliegenthart-wine	5	ci	28.80	39	133	-0.083	0.274
Vliegenthart-beer	6	ci	0.00	302	1269	0.000	NA
Vliegenthart-beer	6	ci	6.25	129	436	-0.223	0.157
Vliegenthart-beer	6	ci	18.75	19	49	-0.010	0.351
Vliegenthart-beer	6	ci	30.00	15	41	-0.261	0.417

id, numeric value only. Type, cc(case control); ir(incidence rate); ci(cumulative incidence). Shim SR [2].

Table S2. The sample data for continuous outcomes

author	id	dose	y	sd	n
Cutler 2006	1	0	5.30	18.31	85
Cutler 2006	1	2	8.23	18.32	92
Cutler 2006	1	5	10.60	18.31	89
Cutler 2006	1	10	11.30	18.32	94
McEvoy 2007	2	0	2.33	26.10	107
McEvoy 2007	2	10	15.04	27.60	103
McEvoy 2007	2	15	11.73	26.20	103
McEvoy 2007	2	20	14.44	25.90	97
Kane 2002	3	0	2.90	24.28	102
Kane 2002	3	15	15.50	26.49	99
Kane 2002	3	30	11.40	22.90	100
Potkin 2003	4	0	5.00	21.14	103
Potkin 2003	4	20	14.50	20.16	98
Potkin 2003	4	30	13.90	20.88	96
Study 94202	5	0	1.40	25.73	57
Study 94202	5	2	11.00	25.00	51
Study 94202	5	10	11.50	25.20	51
Study 94202	5	30	15.80	28.51	54

id, numeric value only. Shim SR [2]
