# Supplementary material for: Dose-response meta-analysis: application and practice using the R software
Source: Epidemiol Health. 2019 Mar 28;41:e2019006. doi: 10.4178/epih.e2019006 (PMC6635664; doi:10.4178/epih.e2019006)
Supplement: Supplementary file 2 [file epih-41-e2019006-supplementary2.pdf]

Volume: 41, Article ID: e2019006

https://doi.org/10.4178/epih.e2019006

<방법론>

용량-반응 메타분석: R을 활용한 적용과 실제

심 성 률[1,2], 이 종 후[3]

[1]고려대학교 의과대학 예방의학교실, [2]순천향대학교 서울병원 비뇨의과학연구소, [3]제주대학교 의과대학 내과

Dose-response meta-analysis: application and practice using R software

Sung Ryul Shim, MPH, PhD[1,2], Jonghoo Lee, MD, PhD[3]

[1]Department of Preventive Medicine, Korea University College of Medicine, Seoul, Korea, [2]Urological Biomedicine Research Institute, Soonchunhyang University Hospital, Seoul, Korea, [3]Department of Internal Medicine, Jeju National University Hospital, Jeju National University School of Medicine, Jeju, Korea

교신저자: 심성률

sungryul.shim@gmail.com

서울시 성북구 안암로 145

Volume: 41, Article ID: e2019006

https://doi.org/10.4178/epih.e2019006

Abstract

The objective of this study was to describe general approaches of dose-response meta-analysis that are available for quantitative synthesis of data using R software.

We conducted a dose-response meta-analysis using two types of data that included difference in means in continuous data and odds ratio in binary data. The package commands of R software were doseresmeta for overall effect sizes that were separated linear model, quadratic model, and restricted cubic split model in order to well understanding. The effect size according to dose and test for linearity were demonstrated and interpreted through analyzing one- and two-stage DRMA. Especially authors stressed several flexible modellings of exposure for pooling of study-specific trends and graphical presentation of the dose-response trend.

This study focused on the practical methods of dose-response meta-analysis rather than theoretical concepts for Korean researchers who were non-majored in statistics. Through this study, authors hope that many Korean researchers will use R software to perform a dose-response meta-analysis more easily and that related research will be activated.

Keywords: Meta-analysis, Dose-response, Quadratic model, Restricted cubic spline, Non-linearity, dosresmeta.

Volume: 41, Article ID: e2019006

https://doi.org/10.4178/epih.e2019006

서론

용량-반응 메타분석(dose-response meta-analysis)은 기본적으로 질병의 위험도와 노출 용량(exposure dose)과의 관계를 밝혀내는 것이다 [1-3].

메타분석 대상 연구들에서 통계량을 추출할 때 약물의 용량에 따라 효과크기가 여러 개 제시되어 있을 경우 연구자는 하나의 연구에서 하나의 효과크기만을 사용할지 또는 모든 용량별 효과크기를 포함해서 분석해야 할지 고민에 빠지게 된다. 전자를 선택하면 연구를 시작하기도 전에 선택편향(selection bias)에 빠지게 될 것이고 후자를 선택하고 싶으나 적절한 메타분석 방법이 소개되어 있지 않다.
따라서 이때 가장 적절한 통계방법이 바로 용량-반응 메타분석이며 특히 노출량을 세 범주(categorical data) 이상으로 분류하여 위험도를 제시한 경우, 노출량에 따른 위험도의 경향(trend)를 보여줄 수 있어 매우 유용한 분석 방법이라고 할 수 있다 [3].

더불어 최근 용량-반응 메타분석을 위한 다양한 분석도구가 개발 되어졌으며 특히 Nicola Orsini가 개발한 two-stage 방법을 응용한 R의 "dosresmeta" 패키지와 STATA의 "drmeta"는 이전까지는 다루지 못했던 연속형 자료까지 분석이 가능하다 [4].

본 연구는 저자가 수행했던 메타분석 선행연구들을 [1-3] R 소프트웨어를 이용해서 다시 풀어낸 것이다. 또한 본 연구는 용량-반응 메타분석을 실행을 위한 R의 기본적인 명령어 사용법과 이를 도식화하는 방법에 초점을 맞출 것이기 때문에 용량-반응 메타분석에 대한 사전 지식이 수반되어야 한다.

1. 용량-반응 메타분석의 이해

1.1. 용량-반응 메타분석 모형

Greenland and Longnecker [5]는 참조범주(referent group)와 각 노출량 구간별(exposure dose level) 위험도의 상관성(correlation)을 일반화 최소제곱모형(generalized least-squares regression)을 활용한 선형성(linear trend) 추정하는 방법을 제시하였다. 또한 Berlin [6]은 용량-반응 다중연구(multiple studies)에서 변량효과 회귀모형(random effect regression)을 적용한 two-stage 메타분석 방법을 제시하였다. two-stage 메타분석이란 first stage에서 개별연구 각각의 회귀계수를 구한다음 second stage에서 개별연구 회귀계수들의 가중평균을 수렴해서 전체 계수를 산정하였다. 하지만 이 방법은 선형성을 가정하였을 때는 적용가능 하지만 비선형(non-linear) 관계에서는 적용되지 않았었다 [3].

따라서 비선형성을 가정한 분석 방법이 요구되어졌으며 용량 구간별로 나누어서 효과크기를 보는 비선형을 가정한 분석이 개발되었다 [7]. 따라서 비선형성 관계에서도 잘 수렴하도록 Liu [8]가 이차분석 모델(quadratic random effect) 모형을 적용하는 방법을 제시하였으며 이를 Orsini [9]가 유동적 비선형 모형을(flexible nonlinear) restricted cubic spline으로 구간화하여 비선형성을 통계적으로 검정하고 이를 그래픽으로 명확히 보여주는 것이 현재의 표준화된 용량-반응 메타분석 방법이다 [1-3].

일반적으로 시간과 용량 의존적인 모델에서는 선형분석 모델보다는 비선형분석 모델인 이차분석 모델(quadratic model), 삼차분석 모델(restricted cubic spline model) 등이 현상을 잘 반영하는 경향이 있다. 따라서 최초 분석을 실시할 때 다양한 모델

Volume: 41, Article ID: e2019006

https://doi.org/10.4178/epih.e2019006

에 대한 검토를 충분히 실시한 후 해당연구에 가장 적합한 모델을 찾아내는 노력이 필요하다.

1.2. 노출용량 결정하기

용량-반응 연구들을 취합할 때 특정 용량을 정확히 제시하고 있는 경우는 거의 없을 것이다. 대부분은 용량을 중위수(median)나 구간별로 제시한다. 중위수로 제시할 경우는 그대로 사용이 가능하지만 구간별로 제시한 경우 연구자는 이를 특정 용량으로 설정해 주어야한다.

기본적인 방법은 다음과 같다.

첫째, 참조범주(reference)의 용량은 0이다 (예, OR이 1인 경우). 둘째, 가장 작은 용량범주의 앞이 열려 있으면 시작을 0으로 간주하고 해당 범주의 중위수를 용량을 한다 (예, <10일 경우 5). 셋째, 구간 범주가 앞과 뒤 수치로 닫혀 있으면 중위수를 용량으로 한다 (예, 10 ~ 20일 경우 15). 넷째, 가장 큰 용량범주의 뒤가 열려 있으면 바로 전 구간 범주의 중위수에서 앞 수치를 뺀 값(15-10=5)을 마지막 범주에 더하여 용량으로 한다 (예, >20일 경우 25) [1-3].

R의 “dosresmeta” 패키지를 이용한 용량-반응 메타분석

Figure 1은 용량-반응 메타분석의 흐름을 나타낸다. 최초 자료 코딩시 해당 함수에 적합하도록 변수명을 수정해야하며, 선형성을 가정한 분석과 비선형성을 가정한 분석을 동시에 실행 후 보고한다.

R에서 용량-반응 메타분석을 실행하려면 "dosresmeta" 패키지가 필요하며 다변량메타분석 관련 패키지 "mvtnorm", "ellipse", "mvmeta"도 미리 설치한다. 더불어 restricted cubic spline을 위해서 “rms” 패키지도 설치한다.

```
·install.packages("dosresmeta")
·install.packages("mvtnorm")
·install.packages("ellipse")
·install.packages("mvmeta")
·install.packages("rms")
```

또한 R에서 일반 중재 메타분석을 실시하는 패키지 “meta”, “metafor” 그리고 ”rmeta”도 미리 설치해 둔다.

```
·install.packages("meta")
·install.packages("metafor")
·install.packaqes("rmeta”)
```

용량-반응 메타분석 대한 상세한 설명은 각 패키지별 상세 코드, 자료, 참고문헌 등이 있으니 참고하기 바란다 [4].

본문과의 구분을 위하여 명령어 앞에는 ‘·’으로 표시하였다. 명령어가 길어져서 다음 줄로 넘어가더라도 ‘·’ 없으면 앞의 줄에서 이어지는 것이다. 따라서 실제 R 프로그램에 입력시에는 ‘·’는 제외하고 타이핑하여야 한다.

1.1. 이분형(binary data) 예제자료

1) 데이터 코딩 및 불러오기

용량-반응 메타분석을 위한 이분형 예제로서 Alcohol 섭취용량에 따른 심혈관질환의 위험도를 R의 예제 데이터에서 발췌하였다 (Supplementary material Table S1).

자료를 코딩할 때 주의할 점은 id는 숫자로 연구를 구분해야하며 type은 case control, incidence rate, cumulative incidence는 각각 cc, ir, ci 문자로 입력한다.

작업폴더에 넣어둔 예제 파일을 아래의 명령어로 R의 메모리에 불러온다. 한가지 주의할 점은 R에서는 쉼표로 구분된 수치파일(csv)의 형태를 선호하니 Appendix 1을 “drma_bin.csv” 포멧으로 저장해서 지정된 작업폴더에 넣어 두어야한다.

```
·data_bin <- read.csv("drma_bin.csv", header=TRUE)
```

read.csv는 csv파일을 불러오는 함수로서 파일명 “drma_bin.csv"를 불러와서 파일의 첫번째 변수명을 그대로 쓴다는 뜻이다 (header=TRUE). 이렇게 로딩된 파일은 R 메모리에서는 data_bin 이름의 데이터로 저장된다.

■ 원자료 산점도 그래프

모델별 분석에 들어가기 앞서 예제파일의 전체적인 윤곽을 알아보기 위하여 산점도 (scatter plot)을 그려보자.

이분형 예제자료에서 표준오차의 역수를 가중치로 쓰는 것이 직관적으로 이해하기 쉽다. 따라서 data_bin 데이터에 추가로 역표준오차 변수를 만든다.

```
·data_bin$inver_se <- 1/data_bin$se
```

그래프는 일반적으로 사용되는 ggplot2 패키지를 사용한다.

```
·library(ggplot2)
·ggplot(data_bin, aes(dose, logrr, size=inver_se)) + geom_point(shape=1, colour="black") +
        scale_size_area(max_size=20)
```

ggplot2 패키지를 메모리에 로딩한 다음 상기 인자들을 차례대로 넣어준다. 전체 세개의 집합이 있는데 첫번째 ggplot 부분은 그래프의 바탕이고 이어지는 geom_point와 scale_size_area는 개별 그래프를 적절히 표현하는 옵션들이다.

첫번째 ggplot 괄호에 데이터명(data_bin)을 넣고 aes에는 가로축변수(dose), 세로축 변수(logrr), 그리고 포인트변수(inver_se)를 넣는다. 이어지는 부분들은 해당 포인트

의 크기와 색깔 등을 설정하는 것으로 여러번 시뮬레이션해보면 개별 요소들을 쉽게 파악할 수 있을 것이다.

Figure 2에 용량(dose)와 효과크기(logrr) 간의 산점도를 나타내었다. 개별 용량별로 효과크기를 볼 수 있으며 원의 크기는 표준오차의 역수이므로 원이 클수록 정밀하고 좋은 연구임을 알 수 있다 (Figure 2).

우측 하단에 있는 두개의 효과크기를 제외하면 전체적으로 증가하는 경향이 있음을 알 수 있다. 따라서 이를 다양한 분석 모델들을 활용하여 검정해보도록 하자.

2) 선형분석 모델 (linear model)

용량-반응 메타분석을 실행하기위해 “dosresmeta” 패키지를 로딩시킨다.

```
·library(dosresmeta)
```

선형성을 가정한 분석에서는 용량변수를 하나의 묶음으로 처리하여 분석을 실시한다.

수집된 대상연구는 개별연구들이 각각 하나씩의 참조범주를 지니는데 two-stage method를 활용하여 개별연구의 회귀계수를 구한다음 이들의 가중평균을 수렴해서 전체 회귀계수를 산정하는 명령어는 다음과 같다.

```
·lin_bin <- dosresmeta(formula = logrr ~ dose, id = id, type = type, se = se, cases = cases,
        n = n, data = data_bin)
·summary(lin_bin)
```

dosresmeta 함수에 여러 인수(argument)를 넣어서 선형분석 모델 lin_bin을 만든다. formula는 용량-반응 메타분석에서 일종의 회귀분석 같은 기능을 한다. 따라서 formula 다음에 종속변수인 logrr을 넣고 ‘~’ 다음에 독립변수인 dose를 위치시킨다. 이후 id, type, se, cases, n을 넣고 이 변수들이 있는 데이터 data_bin을 표시한다.

Summary 명령어를 통해 나온 결과를 console 창에서 살펴보면, 본 분석은 Nicola Orsini가 개발한 two-stage 방법과 REML(Restricted Maximum Likelihood) 모델을 기반으로 계산하였으며 공변량은 Greenland & Longnecker 방법을 사용하였다.

추정된 회귀계수는 -0.0044이며 통계적으로 유의하지는 않았다 (p = 0.4585).

또한 이질성을 확인할 수 있는 Cochrane Q statistics의 p-value(0.0147)와 I^2 값 (64.7%)을 제시하는데 본 선형분석 모델은 이질성이 존재한다는 것을 알 수 있다.

추정된 회귀계수는 로그변환된 상태이기 때문에 해석을 위해서 지수변환 한다.

```
·predict(lin_bin, delta = 1, exp = TRUE)
```

지수변환 후 위험도는 0.996 (95%CI; 0.984, 1.007)이다. 따라서 이를 해석하면 Alcohol을 단위용량 1 섭취했을 때 심혈관질환의 위험도는 0.996배 증가하지만 (또는 0.4% 감소하지만) 95% 신뢰구간이 1을 포함하여 통계적으로 유의하지는 않았다.

■ 선형분석 모델 그래프

Figure 3에 선형분석 모델을 도식화하였다.

```
·dosex_bin <- data.frame(dose = seq(0, 80, 1))
·with(predict(lin_bin, dosex_bin, order = TRUE, exp = TRUE), {
plot(dose, pred, type = "l", col = "blue", ylim = c(0, 2),
ylab = "cardiovascular disease relative risk", xlab = "alcohol consumption, grams/day")
lines(dose, ci.lb, lty = 2)
lines(dose, ci.ub, lty = 2)
})
```

data.frame 함수를 사용하여 용량을 0에서 80까지 1단위로 만들어서 그래프의 X축인 dosex_bin을 설정한다. 본 데이터는 이어지는 이차분석 모델(quadratic model)과 삼차분석 모델(cubic spline model)에서도 동일하게 X축으로 사용되어진다.

그래프를 그리는 명령어는 with이하가 모두 한꺼번에 실행되어야 한다.

https://doi.org/10.4178/epih.e2019006

with는 그래프를 작성할 때 참조할 데이터를 직접 지정하는 것이다. predict함수에 선형분석모델(lin_bin), 그래프의 X축(dosex_bin), 지수변환을 위해서 (exp=TRUE)를 차례대로 넣어준다. 결국 with의 내용은 선형분석모델에 용량 0에서 80까지 1단위로 하나씩 넣어서 나오는 회귀모형의 예측값을 가지고 그래프를 그리겠다는 의미이다.

이어서 plot은 X축에 dose, Y축에 pred에 대응하는 실제 그래프를 그린다. 나머지 명령어는 그림을 보기좋게 하기위한 옵션이니 임의로 추가/제거해보면서 의미를 익히기 바란다.

ylim=c(0, 2)는 Y축을 0에서 2사이만 표시하는 것으로 값을 조절해야 그래프가 적절하게 표현된다. 마지막 두줄 lines는 각각 95% 신뢰구간을 선으로 추가하라는 명령어이다.

앞서 선형분석 모델의 회귀계수를 해석할 때와 동일하게 용량에 따라서 위험도가 감소하는 형태를 나타낸다. 중간에 파란색 실선이 심혈관질환의 위험도이며 아래위 점선이 95% 신뢰구간이다 (Figure 3).

3) 이차분석 모델 (quadratic model)

완전한 선형성을 가정한 분석이 아닌 이차분석 모델을 만들어보자. 간단한 방법은 용량을 제곱 변환하는 것이다.

회귀계수를 산출하는 방법은 앞의 선형분석 모델과 동일하며 모형 설정시 독립변수가 용량과 용량 제곱변환이 추가된다.

```
·quad_bin <- dosresmeta(formula = logrr ~ dose + I(dose^2), id = id, type = type, se = se,
        cases = cases, n = n, data = data_bin)
·summary(quad_bin)
```

dosresmeta 함수를 사용한다. formula에 종속변수인 logrr을 넣고 '~' 다음에 독립변수인 dose와 dose의 제곱을 위치시킨다. 이후 id, type, se, cases, n을 넣고 이 변수들이 있는 데이터 data_bin을 표시한다. 본 모델은 quad_bin으로 설정된다.

전체 모형 적합도는 *p*-value가 0.0414로 유의한 모델이다.

추정된 dose의 회귀계수는 -0.0302이며 통계적으로 유의하였다. (p = 0.022).

또한 이질성을 확인할 수 있는 Cochrane Q statistics의 *p*-value(0.0001)와 I^2 값 (71.5%)로서 이질성이 존재한다는 것을 알 수 있다. 추정된 회귀계수는 로그변환된 상태이기 때문에 해석을 위해서 지수변환 한다.

```
·exp(-0.0302)
```

지수변환 후 위험도는 0.970이다. 따라서 이를 해석하면 Alcohol을 단위용량 1 섭취했을 때 심혈관질환의 위험도는 0.970배 증가하며 (또는 3% 감소하며) 통계적으로 유의하였다.

■ 이차분석 모델 그래프

Figure 4에 이차분석 모델을 도식화하였다.

```
·with(predict(quad_bin, dosex_bin, exp = TRUE), {
  plot(dose, pred, type = "l", ylim = c(0, 15),
    ylab = "cardiovascular disease relative risk", xlab = "alcohol consumption, grams/day")
  lines(dose, ci.lb, lty = 2)
  lines(dose, ci.ub, lty = 2)
})
·points(dosex_bin$dose, predict(lin_bin, dosex_bin, exp = TRUE)$pred, type = "l", lty = 3, col
      = "blue")
```

그래프 명령어에 대한 설명은 선형분석 모델과 동일하다.

다만 ylim=c(0, 15)는 Y축을 0에서 15사이만 표시하는 것으로 값을 적절히 조절해야 그래프가 적절하게 표현된다.

마지막 points는 선형분석 모델도 같은 그림에 표시하기 위한 명령어이다.

가운데 검은색 실선이 위험도이며, 아래위 검은색 점선이 95% 신뢰구간이다. 아래 부분의 파란색 점선은 선형분석 모델의 위험도이다.

이차분석 모델에서의 위험도는 선형분석 모델과 약간 다른 형태를 나타낸다. 용량의 증가에 따라 약 40까지는 유사하지만 이후에는 위험도가 증가하는 경향이 있으며 70을 기점으로는 급격히 상승하는 것을 볼 수 있다. 따라서 이차분석 모델에 따르면 Alcohol 섭취가 고용량 (40이상)이면 심혈관질환의 위험도는 급격히 증가한다는 것을 알 수 있다 (Figure 4).

4) 삼차분석 모델 (restricted cubic spline model)

비선형 모델 중에서 삼차분석 모델을 만들어보자.

회귀계수를 산출하는 방법은 앞의 선형분석 모델과 동일하지만 그에 앞서서 용량구간을 세분화한다.

(1) 용량 구간 분할

용량의 구간을 세분화하여 구간별 용량-반응에 대한 위험도를 보아야 한다. 이때 고려해야 할 것은 개별 연구들의 용량이 동일하지 않으며 또한 용량-반응에 대한 위험도의 관계가 비선형적이어서 용량범주의 구간 설정 문제가 있을 수 있다. 따라서 비선형성 관계에서 용량범주 설정에 관한 최적의 접근은 restricted cubic을 사용하여 구간을 세분화하는 것이 현재까지 개발된 가장 적절한 방법이다 [1-3].

```
·library("rms")
·knots_bin <- quantile(data_bin$dose, c(.05, .35, .65, .95))
```

R에서는 "rms"패키지를 통하여 restricted cubic spline(제한된 삼차 구간분할)이 가능

Volume: 41, Article ID: e2019006

https://doi.org/10.4178/epih.e2019006

하다. quantile함수에 데이터를 지정한다음 벡터형태로 5%, 35%, 65%, 그리고 95% 구간을 분할한다. 용량 구간 분할 개수와 범위는 적절히 조절한다. 네개의 용량을 설정하면 용량구간은 knots_bin은 전체 3개(knots-1개)가 생성된다.

(2) 용량 구간 위험도(회귀계수) 산출

```
·spl_bin <- dosresmeta(formula = logrr ~ rcs(dose, knots_bin), type = type, id = id, se = se,
        cases = cases, n = n, data = data_bin)
·summary(spl_bin)
```

dosresmeta 함수를 사용한다. formula에 종속변수인 logrr을 넣고 '~' 다음에 독립변수를 넣어줄 때 restricted cubic spline(rcs) 함수에 용량과 앞서 분할 해 놓은 용량 3구간을 넣는다. 이후 id, type, se, cases, n을 넣고 이 변수들이 있는 데이터 data_bin을 표시한다. 본 모델은 spl_bin으로 설정된다.

전체 모형 적합도는 *p*-value가 0.0038로 유의한 모델이다.

추정된 용량 구간별 회귀계수를 확인할 수 있는데 선형성 검정을 위해서 임의대로 자료를 분할한 것이니 용량 구간별 위험도를 해석하는 것은 의미가 없다.

(3) 용량 구간 위험도(회귀계수)의 선형성 검정

비선형성을 가정한 분석에서 가장 중요한 부분은 용량 구간별 회귀직선의 기울기가 통계적으로 유의하게 서로 차이가 나는가를 검정하는 것이다. 만약 서로 유의한 차이를 보이지 않는다면 우리는 이것을 선형성이 있다고 판단할 수 있다.

비선형성을 가정한 분석에서 용량구간 세 부분의 기울기가 차이가 나는지 검정해보자.

```
·waldtest(b = coef(spl_bin), Sigma = vcov(spl_bin), Terms = 2:3)
```

Volume: 41, Article ID: e2019006

https://doi.org/10.4178/epih.e2019006

선형성 검정하는 명령어는 waldtest 이다. 용량 세 구간 중 첫번째 구간은 전체 원자료 자체이니 제외하고 용량구간 두번째와 세번째의 joint 기울기를 검정한다.

귀무가설은 H0 : doses2=doses3=0 이다.

따라서 *p*-value가 0.05보다 크면 귀무가설을 받아들여 joint 기울기는 0으로 기울기가 없으며 그리고 두 용량구간의 기울기는 차이가 없으니 선형성이라고 판단한다.

만약 *p*-value가 0.05보다 작으면 귀무가설을 기각하여 joint 기울기는 0이 아니어서 기울기가 있거나 또는 두 용량구간의 기울기는 차이가 있으니 비선형성이라고 판단한다.

Waldtest결과 *p*-value는 0.018로서 본 모델은 비선형성이 있음을 알 수 있다.

■ 삼차분석 모델 그래프

Figure 5에 삼차분석 모델을 도식화하였다.

```
·xref_bin <- 0
·with(predict(spl_bin, dosex_bin, xref_bin, exp = TRUE),{
   plot(get("rcs(dose, knots_bin)dose"), pred, type = "l", ylim = c(0.4, 10),
          ylab = "cardiovascular disease relative risk", xlab = "alcohol consumption,
grams/day", log = "y", bty = "l", las = 1)
   matlines(get("rcs(dose, knots_bin)dose"), cbind(ci.ub, ci.lb), col = 1, lty = "dashed")
})
·points(dosex_bin$dose, predict(lin_bin, dosex_bin, xref_bin, exp = TRUE)$pred, type = "l",
         lty = 3, col = "blue")
```

그래프 명령어에 대한 설명은 선형분석 모델과 동일하다. xref_bin은 용량 참조변수를 특정 수치로 지정하는 것이다. 만들어지는 그래프의 모양에 따라서 참조변수의 수치를 적절히 변경하면 보다 식별력이 좋아질 것이다. 즉, 처음에는 0으로 설정 해보고 그래프의 모양에 따라 조절한다.

ylim=c(0.4, 10)는 Y축을 0.4에서 10사이만 표시하는 것으로 값을 조절해야 그래프가

적절하게 표현된다. 마지막 points는 선형분석 모델도 같은 그림에 표시하기 위한 명령어이다.

가운데 검은색 실선이 위험도이며, 아래위 검은색 점선이 95% 신뢰구간이다. 아래 부분의 파란색 점선은 선형분석 모델의 위험도이다.

삼차분석 모델에서의 위험도는 선형분석 모델과 약간 다른 형태를 나타낸다. 용량의 증가에 따라 약 25까지는 유사하지만 이후에는 위험도가 증가하는 경향이 있으며 40을 기점으로는 상승폭이 증가하는 것을 볼 수 있다. 따라서 삼차분석 모델에 따르면 Alcohol 섭취가 고용량 (30이상)이면 심혈관질환의 위험도는 증가한다는 것을 알 수 있다 (Figure 5).

■ 삼차분석 모델 그래프 조정

Figure 5를 잘 살펴보면 용량이 약 17정도에서 95% 신뢰구간의 폭이 좁아져서 변곡점으로 인식된다. 따라서 이를 수치로 확인해보자

```
·pre_bin <- predict(spl_bin, dosex_bin, exp = TRUE)
·pre_bin$ci.ub - pre_bin$ci.lb
```

Restricted cubic spline 모델의 예측값을 모두 구한다음 신뢰구간의 폭을 계산해보면 결과창 console에 용량 17번째의 값 0.2679로서 가장 폭이 좁은 것을 알 수 있다. 따라서 이를 변곡점으로 설정한 다음 그래프를 다시 그려보자.

```
·xref_bin <- 17
```

용량 참조 변수를 17로 변경한 후 삼차분석 모델 그래프 작성 명령어를 동일하게 실행하면 Figure 6를 만들 수 있다.

참조변수의 수치를 조정한 Figure 6는 용량 17을 기점으로 좌우로 상승하는 J 모양을 나타낸다. 따라서 조금 더 직관적으로 해석할 수 있다.

https://doi.org/10.4178/epih.e2019006

1.2. 연속형(continuous data) 예제자료

1) 데이터 코딩 및 불러오기

용량-반응 메타분석을 위한 연속형 예제로서 약물의 용량에 따른 조현병 (schizophrenia)의 정도를 측정하는 PANSS (positive and negative syndrome scale) 수치를 R의 예제 데이터에서 발췌하였다 (Supplementary material Table S2).

작업폴더에 넣어둔 예제 파일을 아래의 명령어로 R의 메모리에 불러온다. 한가지 주의할 점은 R에서는 쉼표로 구분된 수치파일(csv)의 형태를 선호하니 Appendix 2 를 “drma_con.csv” 포멧으로 저장해서 지정된 작업폴더에 넣어 두어야한다.

```
·data_con <- read.csv("drma_con.csv", header=TRUE)
```

read.csv는 csv파일을 불러오는 함수로서 파일명 “drma_con.csv"를 불러와서 파일의 첫번째 변수명을 그대로 쓴다는 뜻이다 (header=TRUE). 이렇게 로딩된 파일은 R 메모리에서는 data_con 이름의 데이터로 저장된다.

2) 선형분석 모델 (linear model)

용량-반응 메타분석을 실행하기위해 “dosresmeta” 패키지를 로딩시킨다.

```
·library(dosresmeta)
```

앞서 실시한 이분형 예제자료와 설명은 대부분 유사하며 차이가 나는 부분에 집중해서 설명하도록 하겠다.

```
·lin_con <- dosresmeta(formula = y ~ dose, id = id, sd = sd, n = n, covariance = "smd",
        data = data_con)
·summary(lin_con)dosresmeta
```

함수에 여러 인수(argument)를 넣어서 선형분석 모델 lin_con을 만든다. formula는 용량-반응 메타분석에서 일종의 회귀분석 같은 기능을 한다. 따라서 formula 다음에 종속변수인 y를 넣고 '~' 다음에 독립변수인 dose를 위치시킨다. 이후 id, sd, n은 각각 연구, 표준편차, 표본수이다.

covariance에 "smd"를 넣으면 추후 계산을 표준화된 평균의 차이 (smd, standardized mean difference)로 실시하며 "md"를 넣으면 단순 평균의 차이 (absolute mean difference)를 나타낸다.

추정된 회귀계수는 0.0153이며 통계적으로 유의하였다 (p <0.0001). 즉, 약물의 용량이 1 증가하면 조현병 수치는 0.0153 증가하는 것으로 나타났다.

또한 이질성을 확인할 수 있는 Cochrane Q statistics의 p-value(0.5831)와 I^2 값(0%)을 제시하는데 본 선형분석 모델은 이질성이 존재한다는 것을 확인할 수 없었다

■ 선형분석 모델 그래프

Figure 7_a에 선형분석 모델을 도식화하였다.

```
·dosex_con <- data.frame(dose = seq(0, 30, 1))
·with(predict(lin_con, dosex_con, order = TRUE), {
plot(dose, pred, type = "l", col = "blue", ylim = c(0, .6),
ylab = "schizophrenia score", xlab = "medicine dose level, mg/day")
lines(dose, ci.lb, lty = 2)
lines(dose, ci.ub, lty = 2)
})
```

data.frame 함수를 사용하여 용량을 0에서 30까지 1단위로 만들어서 그래프의 X축인 dosex_con을 설정한다. 본 데이터는 이어지는 이차분석 모델(quadratic model)과 삼차분석 모델(cubic spline model)에서도 동일하게 X축으로 사용되어진다.

Volume: 41, Article ID: e2019006

https://doi.org/10.4178/epih.e2019006

그래프를 그리는 명령어는 with이하가 모두 한꺼번에 실행되어야 한다.

with는 그래프를 작성할 때 참조할 데이터를 직접 지정하는 것이다. predict함수에 선형분석모델(lin_con), 그래프의 X축(dosex_con)을 차례대로 넣어준다. 결국 with의 내용은 선형분석모델에 용량 0에서 30까지 1단위로 하나씩 넣어서 나오는 회귀모형의 예측값을 가지고 그래프를 그리겠다는 의미이다.

이어서 plot은 X축에 dose, Y축에 pred에 대응하는 실제 그래프를 그린다. 나머지 명령어는 그림을 보기좋게 하기위한 옵션이니 임의로 추가/제거해보면서 의미를 익히기 바란다.

ylim=c(0, .6)는 Y축을 0에서 0.6사이만 표시하는 것으로 값을 조절해야 그래프가 적절하게 표현된다. 마지막 두줄 lines는 각각 95% 신뢰구간을 선으로 추가하라는 명령어이다.

앞서 선형분석 모델의 회귀계수를 해석할 때와 동일하게 용량에 따라서 위험도가 증가하는 형태를 나타낸다. 중간에 파란색 실선이 조현병 측정 수치이며 아래위 점선이 95% 신뢰구간이다 (Figure 7_a).

3) 이차분석 모델 (quadratic model)

완전한 선형성을 가정한 분석이 아닌 이차분석 모델을 만들어보자. 간단한 방법은 용량을 제곱 변환하는 것이다. 회귀계수를 산출하는 방법은 앞의 선형분석 모델과 동일하며 모형 설정시 독립변수가 용량과 용량 제곱변환이 추가된다.

```
·quad_con <- dosresmeta(formula = y ~ dose + I(dose^2), id = id, sd = sd, n = n, covariance
        = "smd", data = data_con)
·summary(quad_con)
```

dosresmeta 함수를 사용한다. formula에 종속변수인 y를 넣고 '~' 다음에 독립변수인 dose와 dose의 제곱을 위치시킨다. 이후 id, sd, n은 각각 연구, 표준편차, 표본수이다 본 모델은 quad_con으로 설정된다.

전체 모형 적합도는 *p*-value가 0.0001 이하로 유의한 모델이다.

추정된 dose의 회귀계수는 0.0461이며 통계적으로 유의하였다. (p <0.0001). 즉, 약물의 용량이 1 증가하면 조현병 수치는 0.0461 증가하는 것으로 나타났다.

또한 이질성을 확인할 수 있는 Cochrane Q statistics의 *p*-value(<0.0001)와 I^2 값(0%)로서 이질성이 존재한다는 것을 확인할 수 없었다.

■ 이차분석 모델 그래프

```
·with(predict(quad_con, dosex_con, order = TRUE), {
   plot(dose, pred, type = "l", ylim = c(0, .6),
     ylab = "schizophrenia score", xlab = "medicine dose level, mg/day")
   lines(dose, ci.lb, lty = 2)
   lines(dose, ci.ub, lty = 2)
})
·points(dosex_con$dose, predict(lin_con, dosex_con)$pred, type = "l", lty = 3, col = "blue")
```

그래프 명령어에 대한 설명은 선형분석 모델과 동일하다.

다만 ylim=c(0, .6)는 Y축을 0에서 0.6사이만 표시하는 것으로 값을 조절해야 그래프가 적절하게 표현된다.

마지막 points는 선형분석 모델도 같은 그림에 표시하기 위한 명령어이다.

이차분석 모델에서의 위험수치는 선형분석 모델과 약간 다른 형태를 나타낸다. 용량의 증가에 따라 약 20까지는 유사하지만 이후에는 위험도가 감소하는 경향이 있으며 20-25을 기점으로는 급격히 감소하는 것을 볼 수 있다. 따라서 이차분석 모델에 따르면 약물 용량이 20-25 기준으로 상승과 하강을 보이는 포물선 모형이다.

4) 삼차분석 모델 (restricted cubic spline model)

비선형 모델 중에서 삼차분석 모델을 만들어보자.

회귀계수를 산출하는 방법은 앞의 선형분석 모델과 동일하지만 그에 앞서서 용량구간을 세분화한다.

(1) 용량 구간 분할

용량의 구간을 세분화하여 구간별 용량-반응에 대한 위험수치를 보아야 한다.

```
·library("rms")
·knots_con <- quantile(data_con$dose, c(.05, .5, .95))
```

R에서는 "rms"패키지를 통하여 restricted cubic spline(제한된 삼차 구간분할)이가능하다. quantile함수에 데이터를 지정한다음 벡터형태로 5%, 50%, 그리고 95% 구간을 분할한다. 용량 구간 분할 개수와 범위는 적절히 조절한다. 세개의 용량을 설정하면 용량구간은 knots_con은 전체 2개(knots-1개)가 생성된다.

(2) 용량 구간 위험도(회귀계수) 산출

```
·spl_con <- dosresmeta(formula = y ~ rcs(dose, knots_con), id = id, sd = sd, n = n, covariance
        = "smd", data = data_con)
·summary(spl_con)
```

dosresmeta 함수를 사용한다. formula에 종속변수인 y를 넣고 '~' 다음에 독립변수를 넣어줄 때 restricted cubic spline(rcs) 함수에 용량과 앞서 분할 해 놓은 용량 2구간을 넣는다. 이후 id, sd, n을 넣고 이 변수들이 있는 데이터 data_con을 표시한다. 본 모델은 spl_con으로 설정된다.

전체 모형 적합도는 *p*-value가 0.0001 이하로 유의한 모델이다. 추정된 용량 구간별 회귀계수를 확인할 수 있는데 선형성 검정을 위해서 임의대로 자료를 분할한 것이니 용량 구간별 위험수치를 해석하는 것은 의미가 없다.

(3) 용량 구간 위험도(회귀계수)의 선형성 검정

비선형성을 가정한 분석에서 가장 중요한 부분은 용량 구간별 회귀직선의 기울기가 통계적으로 유의하게 서로 차이가 나는가를 검정하는 것이다. 만약 서로 유의한 차이를 보이지 않는다면 우리는 이것을 선형성이 있다고 판단할 수 있다.
비선형성을 가정한 분석에서 용량구간 두 부분의 기울기가 차이가 나는지 검정해보자.

·waldtest(b = coef(spl_con), Sigma = vcov(spl_con), Terms = 1:2)

선형성 검정하는 명령어는 waldtest 이다. 용량구간 첫번째와 두번째의 joint 기울기를 검정한다.
귀무가설은 H0 : doses1=doses2=0 이다.

Waldtest결과 *p*-value는 0.0001이하로서 본 모델은 비선형성이 있음을 알 수 있다. 그러나 본 연속형 예제처럼 restricted cubic spline으로 용량 구간을 세 구간 이하로 분할한 경우 첫번째 구간이 전체 원자료 자체이기 때문에 waldtest를 통한 선형성 해석에 주의를 기울여야한다. 즉, waldtest를 통해서 두 구간의 선형성 검정결과를 받아들일지 아니면 첫번째 구간(원자료 자체)의 회귀계수의 유의성으로 선형성을 판단해야 할지 종합적인 검토가 필요하다.

Volume: 41, Article ID: e2019006

https://doi.org/10.4178/epih.e2019006

■ 삼차분석 모델 그래프

Figure 7_b에 삼차분석 모델을 도식화하였다.

```
·xref_con <- 0
·with(predict(spl_con, dosex_con, xref_con),{
   plot(get("rcs(dose, knots_con)dose"), pred, type = "l", ylim = c(0, .6),
      ylab = "schizophrenia score", xlab = "medicine dose level, mg/day", bty = "l", las = 1)
   matlines(get("rcs(dose, knots_con)dose"), cbind(ci.ub, ci.lb), col = 1, lty = "dashed")
})
·points(dosex_con$dose, predict(lin_con, dosex_con, xref_con)$pred, type = "l", lty = 3, col
         = "blue")
```

그래프 명령어에 대한 설명은 선형분석 모델과 동일하다. xref_con은 용량 참조변수를 특정 수치로 지정하는 것이다. 즉, 처음에는 0으로 설정해보고 그래프의 모양에 따라 조절한다.

ylim=c(0, .6)는 Y축을 0에서 0.6사이만 표시하는 것으로 값을 조절해야 그래프가 적절하게 표현된다. 마지막 points는 선형분석 모델도 같은 그림에 표시하기 위한 명령어이다.

가운데 검은색 실선이 위험도이며, 아래위 검은색 점선이 95% 신뢰구간이다. 아래 부분의 파란색 점선은 선형분석 모델의 위험수치이다.

삼차분석 모델에서의 위험도는 선형분석 모델과 약간 다른 형태를 나타내지만 이차분석 모델과는 매우 유사하다.

용량의 증가에 따라 약 20까지는 유사하지만 이후에는 위험도가 감소하는 경향이 있으며 20-25을 기점으로는 급격히 감소하는 것을 볼 수 있다. 따라서 삼차분석 모델에 따르면 약물 용량이 20-25 기준으로 상승과 하강을 보이는 포물선 모형임을 알 수 있다 (Figure 7_b).

Volume: 41, Article ID: e2019006

https://doi.org/10.4178/epih.e2019006

맺음말

본 연구는 통계학을 전공하지 않은 일반 연구자들도 쉽게 수행할 수 있도록 통계학 이론을 최소화하고 메타분석의 실질적 수행방법에 집중하였다. 즉, 일반 연구자들이 이미 개발되어진 통계방법들을 잘 활용하여 본인의 연구분야에 적합하게 쓰고 해석하기 위함이다.

용량-반응 메타분석을 위한 R 패키지는 매우 강력한 기능들을 다룰 수 있다. 연구자들이 조그만 익숙해진다면 충분히 응용가능한 분석 방법이다. 아울러 본 연구에서 다루지 않았지만 STATA에서 동일한 분석도구인 "drmeta"의 명령어가 있으니 참조하기 바란다.

아울러 본 연구를 통하여 많은 국내 연구자들이 보다 쉽게 메타분석을 수행하고 관련 연구가 활성화되기를 바란다.

Volume: 41, Article ID: e2019006

https://doi.org/10.4178/epih.e2019006

References

1. Hwang SD & Shim SR. Meta-analysisp; from forest plot to network meta-analysis. Seoul: Hannarae publishing co.; 2018 (Korean). ISBN: 9788955662214

2. Shim SR. Dose response meta-analysis using R software. Gyeonggi-do: SDB Lab; 2019 (Korean). ISBN 979-11-965933-3-9.

3. Shim SR, Shin IS, Yoon BH, Bae JM. Dose-response meta-analysis using STATA Software. Journal of Health Informatics and Statistics 2016;41(3):351-358.

4. Dose response "doseresmeta" packages. Available from: https://alecri.github.io/software/dosresmeta.html or https://github.com/alecri/dosresmeta

5. Greenland, S, & Longnecker, M. P. Methods for trend estimation from summarized dose-response data, with applications to meta-analysis. Am J Epidemiol. 1992;135(11):1301–1309.

6. Berlin JA, Longnecker MP, Greenland S. Meta-analysis of epidemiologic dose-response data. Epidemiology 1993;4(3):218-228.

7. Greenland, S. Avoiding power loss associated with categorization and ordinal scores in dose-response and trend analysis. Epidemiology. 1995;6(4):450–454.

8. Liu, Q., Cook, N. R. & Bergstrom, A. A two-stage hierarchical regression model for meta-analysis of epidemiologic nonlinear dose-response data. Comput Stat Data Anal. 2009;53(12):4157–4167.

9. Orsini, N. & Greenland, S. A procedure to tabulate and plot results after flexible modeling of a quantitative covariate. Stata Journal. 2011;11:1–29.

Volume: 41, Article ID: e2019006

https://doi.org/10.4178/epih.e2019006

Figure legends

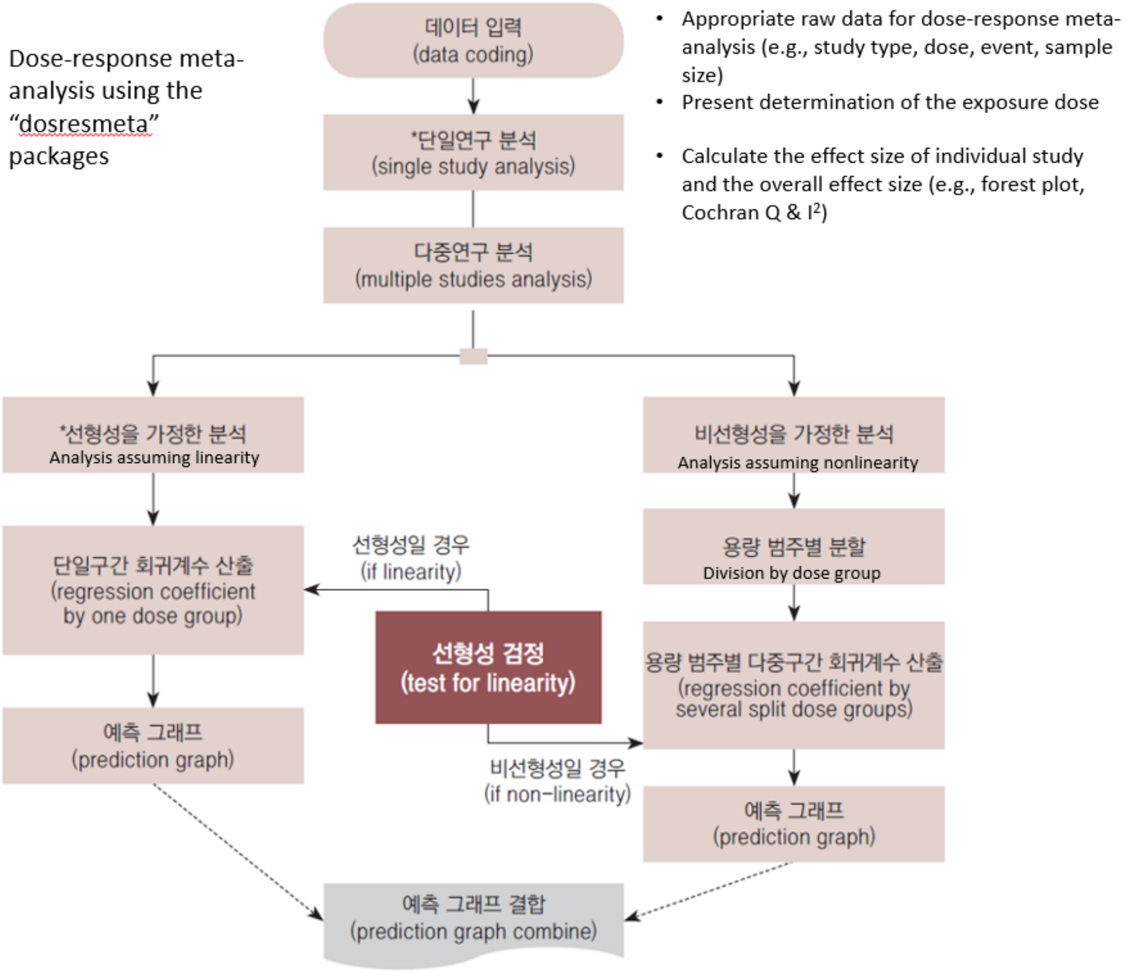

Figure 1. Flow chart of dose-response meta-analysis using R "dosresmeta" package.

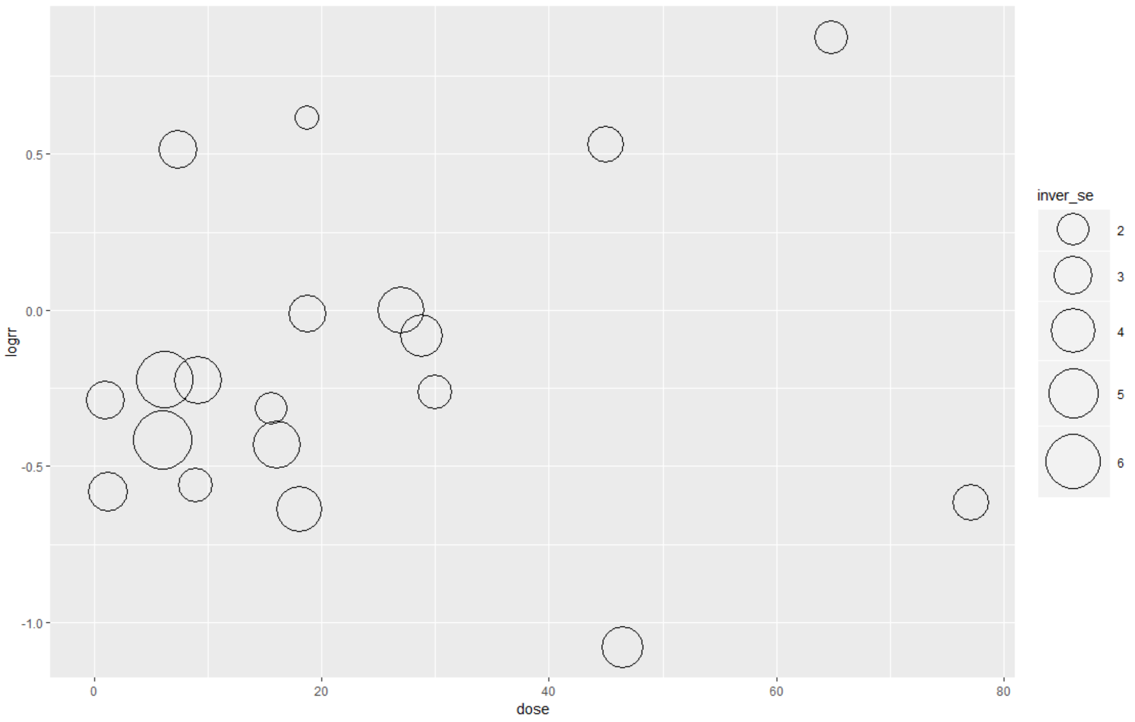

Figure 2. Scatter plot of binary sample data.

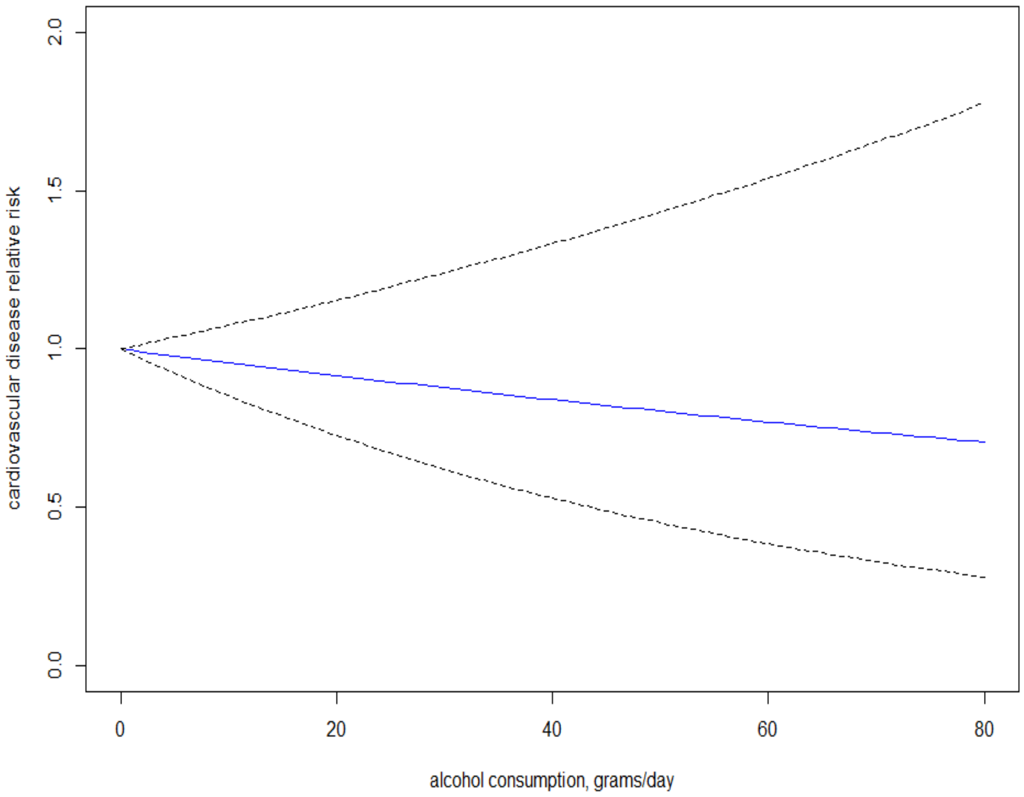

Figure 3. Linear model of binary sample data.

Volume: 41, Article ID: e2019006

https://doi.org/10.4178/epih.e2019006

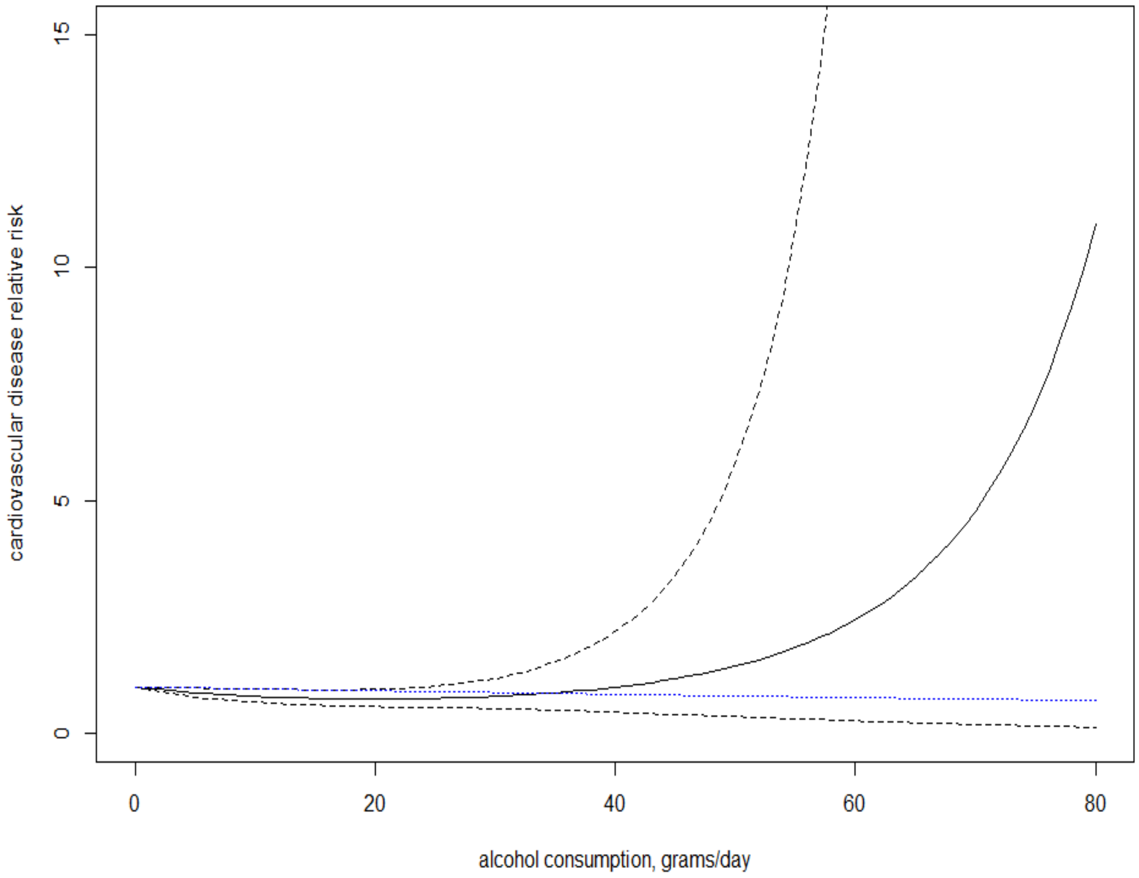

Figure 4. Quadratic spline model of binary sample data (ref=0)..

Volume: 41, Article ID: e2019006

https://doi.org/10.4178/epih.e2019006

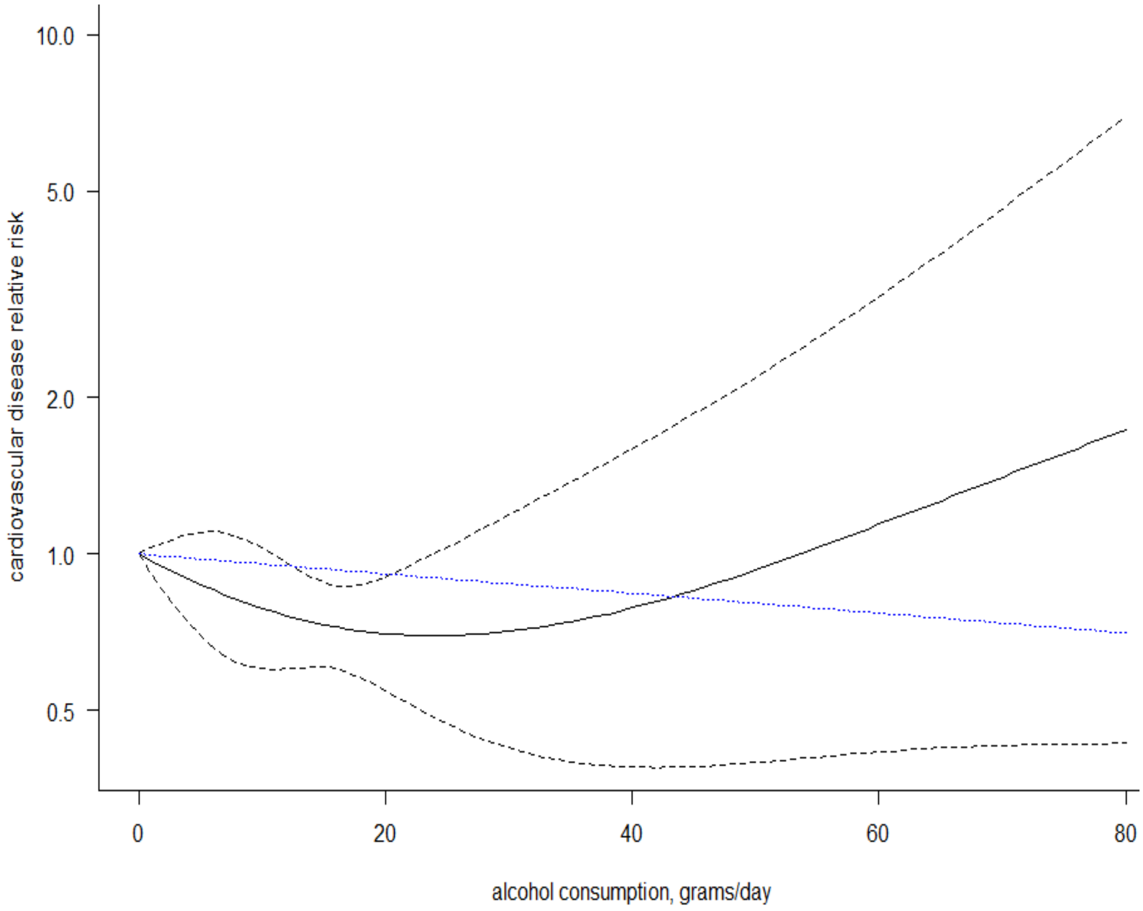

Figure 5. Restricted cubic spline model of binary sample data (reference=0).

Volume: 41, Article ID: e2019006

https://doi.org/10.4178/epih.e2019006

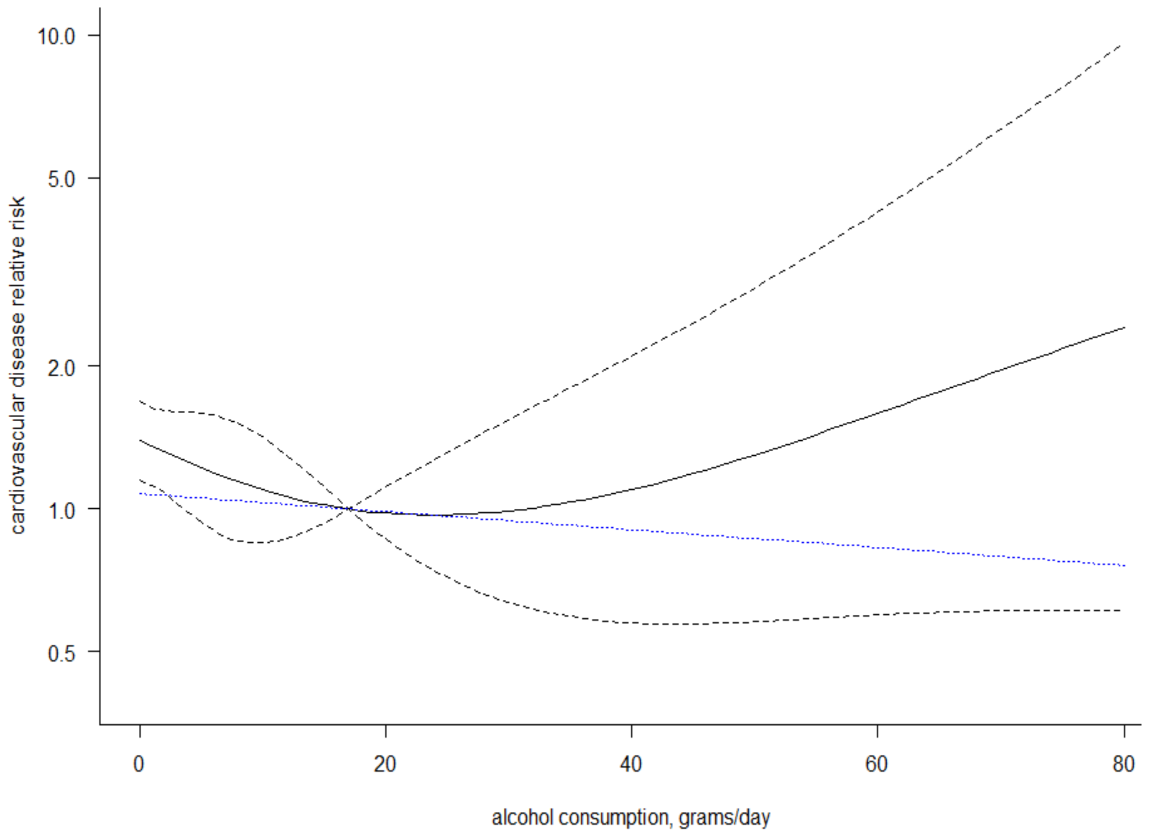

Figure 6. Restricted cubic spline model of binary sample data (reference=17).

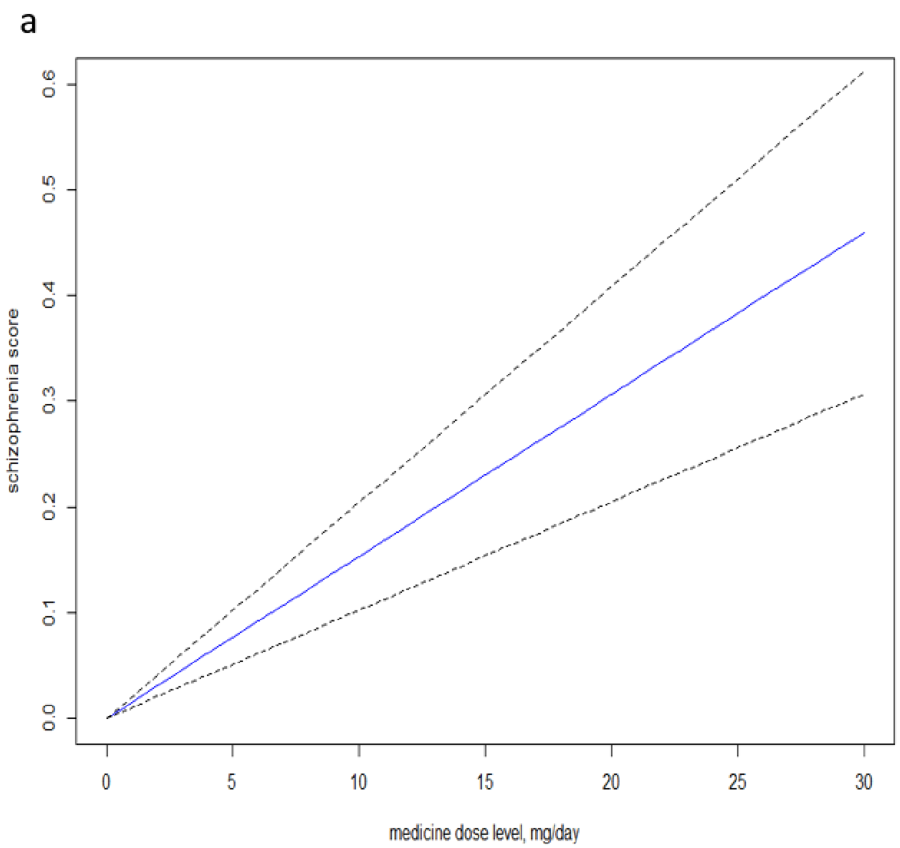

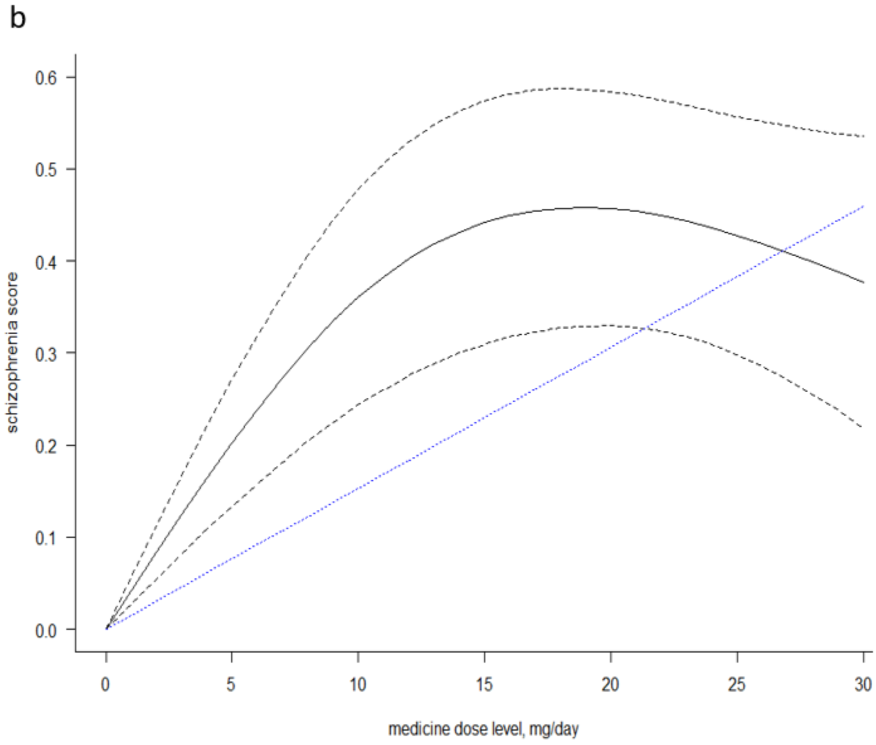

Figure 7. a, Linear model. b, Restricted cubic spline model of continuous sample data (ref=0).
